# Long-term care use, hospitalizations and mortality during COVID-19 in Finland and Sweden: A nationwide register-based study in 2020

**DOI:** 10.1177/14034948241235730

**Published:** 2024-03-13

**Authors:** Pierre-Olivier Blotière, Géric Maura, Jani Raitanen, Jutta Pulkki, Leena Forma, Kristina Johnell, Mari Aaltonen, Jonas W. Wastesson

**Affiliations:** 1 Department of Medical Epidemiology and Biostatistics, Karolinska Institutet, Stockholm, Sweden; 2 Faculty of Social Sciences (Health Sciences) and Gerontology Research Centre (GEREC), Tampere University, Tampere, Finland; 3 UKK Institute for Health Promotion Research, Tampere, Finland; 4 Finnish Institute for Health and Welfare, Helsinki, Finland; 5 Laurea University of Applied Sciences, Vantaa, Finland; 6 Aging Research Centre, Department of Neurobiology, Care Sciences and Society, Karolinska Institutet & Stockholm University, Sweden

**Keywords:** Aged, COVID-19, hospitalization, long-term care, mortality, register

## Abstract

**Aim:**

To describe long-term care (LTC) use in Finland and Sweden in 2020, by reporting residential entry and exit patterns including hospital admissions and mortality, compared with the 2018–2019 period and community-living individuals.

**Methods:**

From national registers in Finland and Sweden, all individuals 70+ were included. Using the Finnish and Swedish study populations in January 2018 as the standard population, we reported changes in sex- and age-standardized monthly rates of entry into and exit from LTC facilities, mortality and hospital admission among LTC residents and community-living individuals in 2020.

**Results:**

Around 850,000 Finns and 1.4 million Swedes 70+ were included. LTC use decreased in both countries from 2018 to 2020. In the first wave (March/April 2020), Finland experienced a decrease in LTC entry rates and an increase in LTC exit rates, both more marked than Sweden. This was largely due to short-term movements. Mortality rates peaked in April and December 2020 for LTC residents in Finland, while mortality peaked for both community-living individuals and LTC residents in Sweden. A decrease in hospital admissions from LTC facilities occurred in April 2020 and was less marked in Finland versus Sweden.

**Conclusions:**

During the first wave of the pandemic mortality was consistently higher in Sweden. We also found a larger decrease in LTC use and, among LTC residents, a smaller decrease in hospital admissions in Finland than in Sweden. This study calls for assessing the health consequences of the differences observed between these two Scandinavian countries as part of the lessons from the COVID-19 pandemic.

## Background

In 2020, the COVID-19 pandemic, with its associated restrictions, significantly impacted the daily lives and care of older people worldwide and affected the availability, demand and provision of health and social care services [1]. People over the age of 70 years accounted for the majority of all COVID-19 deaths in Finland [2] and Sweden [3] in 2020. In both countries, almost half of all COVID-19 deaths occurred in long-term care (LTC) facilities [2,4,5]. However, in the first year of the pandemic, the societal spread of infection was much smaller in Finland than in Sweden, with 9.9 and 84.3 COVID-19 deaths per 100,000 respectively [6]. To date, few national studies have focused on describing how the pandemic played out in the LTC setting.

Both Finland and Sweden rely on universal health and social care service systems, in particular for the care for older people [7]. In Sweden, home care and LTC are organized by the municipalities, whilst health care is the responsibility of regions. In Finland, primary health care, home care and LTC were organized by municipalities and secondary health care by hospital districts until January 2023, when they were transferred to the wellbeing services counties [7].

The two countries adopted partly different strategies to mitigate the spread of the virus [8–10]. Overall, Finland adopted stricter preventative measures at the population level compared with Sweden [11]. In Sweden early response was built largely on voluntary adherence to recommendations from public authorities [10,12]. Early in the pandemic, the Finnish Government declared a state of emergency and announced that the focus was on increasing healthcare capacity to care for severe COVID-19 cases. Simultaneously, non-urgent activities were reduced [13]. Regarding care for older people, several guidelines and recommendations were published in Finland between March and May 2020, including restrictions for family members to visit their relatives in the LTC facilities [14]. Another guideline was to avoid transfers between care sites, such as between LTC facilities and hospitals [8]. In Sweden, on 10 March 2020, the population was advised to avoid unnecessary visits to hospital inpatients or LTC facilities and by the end of March 2020, both private LTC providers and municipalities had banned visits to LTC facilities until 1 October [10]. National Swedish guidelines on how to reduce the spread of COVID-19 in LTC facilities were published in early 2021 May [9].

The objective of this study was to describe, among people aged 70 years or older, LTC use in Finland and Sweden in 2020, by reporting residential entry and exit patterns including hospital admissions and mortality, compared with previous years (2018–2019) and with older adults living in the community, based on national registers data from the two countries.

## Methods

### Data sources

This descriptive study was based on comprehensive national registers from Finland and Sweden: the Swedish total population register [15], which contains sociodemographic data and data on life events including migration; Finnish population data through Statistics Finland, including sociodemographic data [16], the Finnish Care Register for Health Care [17] and the Swedish Patient Register [18], which both contain data on hospital admissions, including admission dates and diagnoses; the Finnish Care Register for Social Welfare [19] and the Swedish Social Service Register [20], which contain in particular monthly data on LTC use. In this article, LTC residents refers to round-the-clock LTC facility residents while living in the community refers to older people not living in LTC facilities. Last, the Finnish and Swedish Causes of Death Registers [21] contain data on death certificates, including the date of death and the underlying cause of death. Both hospital diagnoses and causes of death are coded according to the International Classification of Diseases, 10th Revision. These datasets were individually linked using a unique personal identity number in both Finland and Sweden [22].

For Finland, permission to access the register data was obtained from the register maintainers (Finnish Institute for Health and Welfare and Statistics Finland) in the remote access system Fiona. The research plan was approved by the Pirkanmaa Hospital District Ethics Committee (9/2020 ETL code R2012TR). For Sweden, all data were pseudonymized to the researchers and ethical approval was granted from EPM (DNR 2016/1001–31/4, 2020–03525; 2021–02004).

### Study population

The monthly study population included all individuals 70+ at the beginning of each year (2018, 2019 and 2020) and alive at the beginning of each month. For Sweden only, individuals were required to be living in the country at the beginning of each month as data on migrations were available. The social service register has poor coverage for few municipalities in Sweden [20]. Hence, we excluded individuals living in nine out of 290 municipalities which did not report monthly data on LTC use at least once between 2018 and 2020.

### Outcomes

We studied the changes in the monthly rates of (A) entry into LTC facility, (B) exit from LTC facility, (C) mortality among older adults living in the community, (D) mortality among LTC residents, (E) hospital admission among older adults living in the community and (F) hospital admission among LTC residents.

For outcome A, the monthly population defined above was further restricted to individuals not registered in a LTC facility in the previous month and for outcome B to individuals registered in a LTC facility in the given month.

For outcome B, individuals also had to be alive at the end of the month to avoid considering death as an exit from LTC facility. For the other outcomes, LTC residents and community-living individuals were defined as registered and not registered, respectively, in a LTC facility in the given month. These monthly populations constituted the denominator of the rates.

The numerator of the rates consisted of individuals from the outcome-specific monthly populations defined above who: were registered in a LTC facility in the given month for outcome A; were not registered in a LTC facility in the following two months for outcome B; died in the given month for outcomes C and D; and were hospitalized for at least one night in the given month for outcomes E and F.

### Statistical analyses

We used a common protocol, and we conducted the analyses separately in both countries on datasets generated from national registry data.

We first described the study populations according to year, sex, age and LTC use. For 2020 only, we reported the proportion of annual and monthly COVID-19 cases, defined as deaths with U071 (COVID-19, virus identified) or U072 (COVID-19, virus not identified) as the underlying cause of death, or hospitalizations with U071 or U072 as the main discharge diagnosis. This allowed us to describe the timing and magnitude of the COVID-19 waves in 2020 to better interpret the main outcomes.

We reported the unstandardized and sex- and age-standardized monthly rates of the six outcomes, using the total of the Finnish and Swedish study populations in January 2018 (February 2018 for the first outcome) as the standard population.

#### Sensitivity analyses

To test the impact of potential short-term stays, we modified the definitions of entry into and exit from LTC used in the main analyses:

1. For outcome A (entry), we considered two then three consecutive months (instead of one in the main analysis) to define entry into LTC facility.
2. For outcome B (exit), we also varied the number of consecutive months with no registration into LTC facility, as one and three (instead of two) to define exit from LTC facility.

As only monthly LTC registration data were available in Sweden, the hospitalization (for which the exact date was available) may have happened before the actual date of LTC entry. Therefore, for outcome F, we restricted the study population to individuals registered in a LTC facility in both the given and the previous month, instead of the given month only. This prevented us from wrongly considering these hospitalized individuals as LTC residents.

Finally, to assess the consistency in the reporting of LTC use by the Swedish municipalities, we excluded individuals living in municipalities for which the monthly number of LTC residents was at least 50% (or 25%) lower or higher than the mean between January 2018 and December 2019 at least once.

## Results

At the beginning of each year, the study population was composed of around 850,000 individuals in Finland, and around 1.4 million 70+ individuals in Sweden (Table I), after the exclusion of around 74,000 individuals living in Swedish municipalities which did not report monthly data on LTC use regularly, which represented 5.0% of the older adults living in Sweden (Supplemental material Table I online). The study population was slightly older in Finland than in Sweden, and the proportions of women and persons living in LTC facilities were higher in Finland than in Sweden. A decreasing trend in LTC use was observed in both Finland and Sweden from 2018 to 2020.

**Table I.** Population characteristics and COVID-19 cases in Finland and Sweden.

	Finland			Sweden		
	2018	2019	2020	2018	2019	2020
*N* ^a^	810,149	843,398	874,889	1,371,685	1,410,306	1,447,213
Women	472,099 (58.3%)	488,488 (57.9%)	504,206 (57.6%)	752,530 (54.9%)	770,658 (54.6%)	788,256 (54.5%)
Age (mean ± STD), in years	79.0 ± 6.5	78.9 ± 6.5	78.9 ± 6.5	78.0 ± 6.5	78.0 ± 6.5	78.0 ± 6.4
Age categories, in years						
70–74	307,971 (38.0%)	331,096 (39.3%)	350,043 (40.0%)	528,395 (38.5%)	535,117 (37.9%)	531,860 (36.8%)
75–79	207,343 (25.6%)	209,498 (24.8%)	212,365 (24.3%)	357,006 (26.0%)	379,911 (26.9%)	406,695 (28.1%)
80–84	148,687 (18.4%)	155,212 (18.4%)	161,488 (18.5%)	239,162 (17.4%)	248,144 (17.6%)	259,069 (17.9%)
85–89	96,803 (11.9%)	96,057 (11.4%)	96,876 (11.1%)	154,723 (11.3%)	153,857 (10.9%)	155,133 (10.7%)
90+	49,345 (6.1%)	51,535 (6.1%)	54,117 (6.2%)	92,399 (6.7%)	93,277 (6.6%)	94,456 (6.5%)
LTC residents^b^	90,842 (11.2%)	90,416 (10.7%)	86,256 (9.9%)	100,877 (7.4%)	99,637 (7.1%)	98,459 (6.8%)
COVID-19 cases, – death or hospitalization	NA	NA	1004 (0.1%)	NA	NA	17,492 (1.2%)
COVID-19 deaths	NA	NA	491 (0.1%)	NA	NA	8078 (0.6%)
COVID-19 hospitalizations	NA	NA	711 (0.1%)	NA	NA	13,390 (0.9%)

Figures are *n* (%), except for age (mean ± std).

a For each year, the population considered in this table is the study population in January.

b An individual is considered as a LTC resident if he/she is registered for at least one night/day in a LTC facility in the year considered.

LTC: long-term care; NA: not available

**Figure 1.**
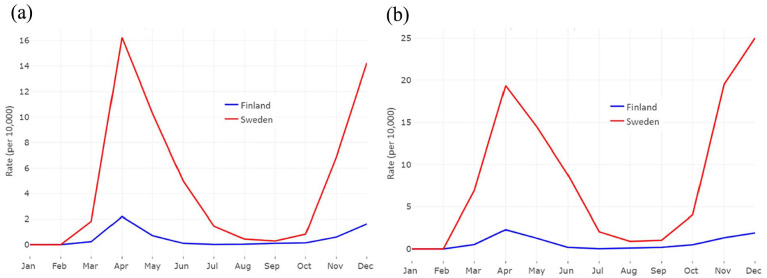
Monthly COVID-19-related mortality (a) and hospitalization (b) rates in Finland and Sweden in 2020.

The timing of the COVID-19 waves among older people was the same in Finland and Sweden, but the magnitude was larger in Sweden (Figure 1). The number of COVID-19 cases in Finland was one-tenth compared with Sweden (Table I).

**Figure 2.**
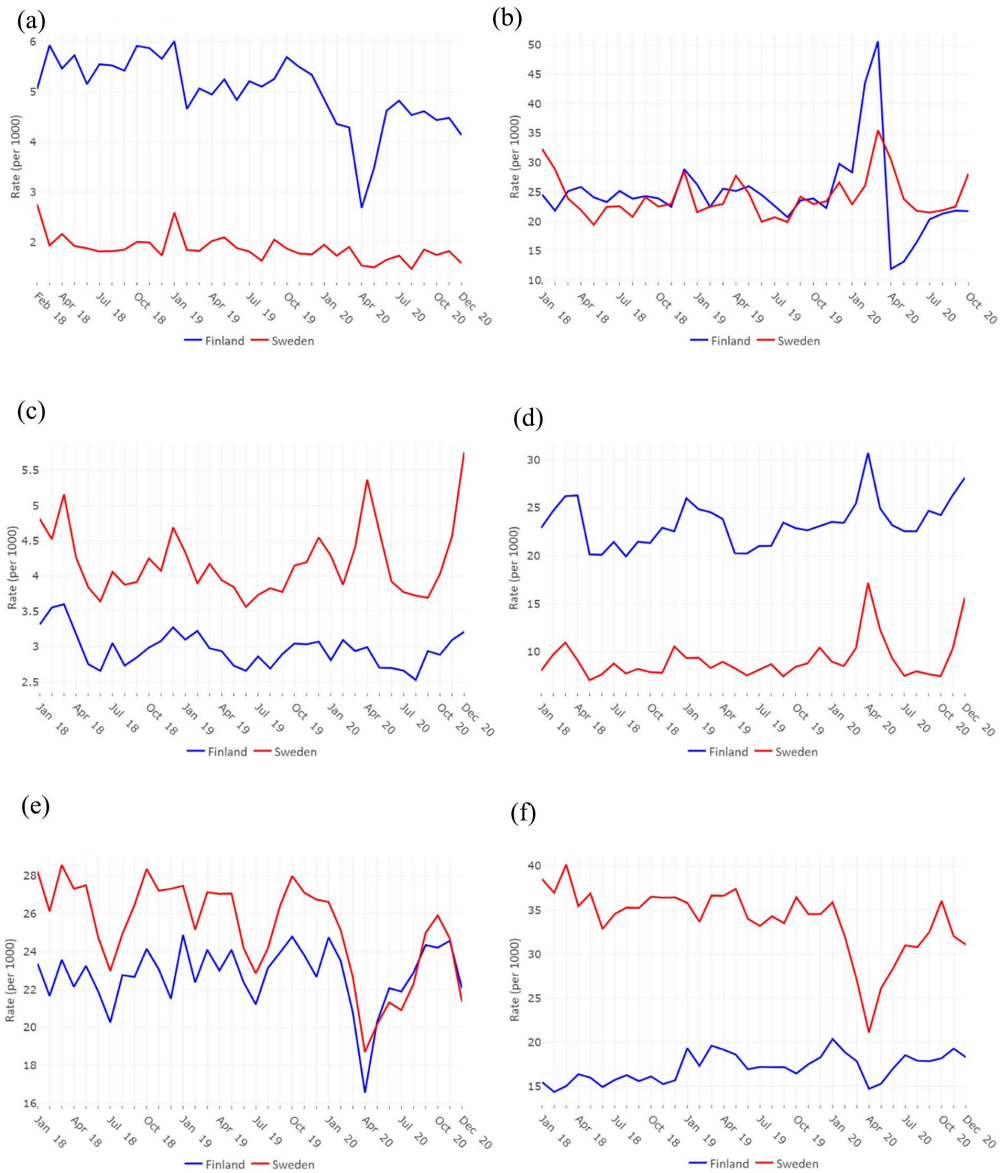
Standardized monthly rates for the main six outcomes for Finland (blue lines) and Sweden (red lines): entry in a long-term care (LTC) facility (a*), exit from a LTC facility (b*), mortality among community-dwellers (c), mortality among LTC residents (d), hospital admission among community-dwellers (e) and hospital admission among LTC residents (f). *As no data on LTC use is available before 2018-01-01 for Finland and after 2020-12-31, outcome A was calculated from February 2018 and outcome B was calculated until October 2020.

Monthly entry rates in LTC facilities were two to three times as high in Finland as in Sweden, while exit rates were quite similar over the study period. Finland experienced a much more marked decrease in entry rates in April 2020 (−37.4% compared with −19.5% in Sweden, March to April) and a much more marked increase in exit rates in March 2020 than Sweden (Figure 2(a) and (b)).

Monthly mortality rates among LTC residents were two to three times as high in Finland as they were in Sweden over the study period. In Sweden, mortality rates peaked in April and December 2020 for both community-living and LTC residents, while mortality rates peaked in April and December 2020 only for LTC residents in Finland, with a lower magnitude (Figure 2(c) and (d)).

Monthly hospital admission rates were similar in Finland and Sweden among older adults living in the community over the study period, while they were around twice as high in Sweden as they were in Finland among LTC residents, although the rates display a convergence over the study period. A large decrease in hospital admissions among persons living in the community in April 2020 and a smaller one in December 2020 were observed for both countries. For LTC residents, the decrease in April 2020 was less marked in Finland than in Sweden (Figure 2(e) and (f)).

There were almost no differences between sta-ndardized and unstandardized rates (Supplemental Figure 1).

### Sensitivity analyses

Considering a stricter definition of entry into and exit from LTC decreased the level of entry and exit rates for Finland while it did not change the results (outcomes A and B) for Sweden (Supplemental Figure 2(b)).

Considering a stricter definition of LTC residents led to similar results regarding the hospitalization rates among LTC residents (outcome F) for both countries (Supplemental Figure 2(f)).

Finally, considering stricter exclusion criteria regarding the consistency in the reporting of LTC use did not change the level and trends of the rates (Supplemental Figure 3).

## Discussion

In this descriptive study of the first year of the COVID-19 pandemic in Finland and Sweden, we confirmed that: (i) there was almost no increase in mortality rates among older adults living in the community in Finland and a high increase in Sweden, especially in April and December 2020 [6]; (ii) in LTC facilities, both countries experienced an increase in mortality rates, with a higher increase in Sweden [23]; (iii) there was a decrease in hospital admissions around April 2020 [1]. In addition, we found substantial differences in the patterns of LTC use between Finland and Sweden during the first year of the COVID-19 pandemic, with much more intense changes in entry and exit rates in Finland. We also showed that the decrease in hospital admissions among LTC residents was smaller in Finland than in Sweden.

From an international perspective, Finland and Sweden have similar systems for care for older people [7]. Nonetheless, they were differently affected by COVID-19 in the first year of the pandemic [6]. To our knowledge, no comparison of the Finnish and Swedish LTC use had been done before the pandemic. We observed three main differences in LTC use between Finland and Sweden before the pandemic (years 2018 and 2019). First, Finland had higher entry rates in LTC facilities. Our sensitivity analysis, where short-term residents were excluded, suggests that Finland had a higher use of respite and short-term placements, which often are related to the Finnish official informal care support system. Second, the mortality rates were higher in Finnish LTC facilities. Dying in LTC facilities has become more common in Finland in recent decades [24]. It is possible that older people in Finland move to LTC facilities at a later stage than in Sweden, and, when they move there, they are less likely to be hospitalized at the end of life than in Sweden. These latter two points may explain Finland’s higher mortality rate in LTC facilities. The third difference is the lower hospitalization rate in Finnish compared with Swedish LTC facilities [25]. A possible explanation for this is the closer organizational ties between healthcare and long-term care in Finland [7] and the result of the efforts made by Finland to reduce avoidable hospitalizations in end-of-life care [26].

During 2020, at the beginning of the COVID-19 pandemic, a significant decrease in the entry rate into LTC facilities and increase in exit rates was found in Finland (in both absolute and relative terms), whereas the entry and exit rates remained rather unchanged in Sweden. In the sensitivity analysis where only entries and exits for at least three months were considered, the substantial changes observed in Finland were reduced to levels more similar to Sweden. Therefore, short-term movements seem to explain a large part of the rapid changes observed in Finland. A short-term movement in Finland is often due to the leave of informal carers. Those who have the official informal care agreement are entitled to at least two days’ leave in a month from their informal carer duties. In this case, the person needing care can access the LTC facility for short-term care. Sweden does not have a national system for carer leave. In line with what we observed, a large number of Finnish municipalities closed the option of short-term respite care in March 2020 [14]. Alternatively, the more rapidly changing rates in Finland may reflect an attempt to avoid LTC facilities in response to the media coverage of the nursing home crisis [27].

Avoiding unnecessary visits to healthcare and LTC facilities was encouraged in both Finland and Sweden during the spring of 2020, including the postponement of routine visits and screening to avoid overburdening the healthcare systems [10,28,8]. Internationally, a decline in healthcare services use has been reported [1]. Expectedly, we found that hospitalization rates decreased in both the community and LTC facilities in Sweden, whereas it only decreased among Finnish older adults living in the community. A potential reason why hospital admissions did not decline among Finnish LTC residents can be the lower burden of COVID-19 cases during 2020 and that Finland managed to sustain healthcare for older adults with large needs. The potential trade-off between avoiding healthcare visits and creating a care debt, that is, the necessary care is not received on time, was potentially underestimated in the early phase of the pandemic, especially in Sweden [28]. Our data, only covering the first wave of the pandemic, do not support a full-scale investigation of which kind of hospitalizations were avoided, which would provide important information about medical decision-making during the pandemic.

The observed mortality rates confirm previously published patterns of COVID-19 mortality in Finland and in Sweden [6]. Sweden displayed much elevated mortality rates during the two waves in 2020, in both community-living older adults and LTC residents. In Finland, the mortality rates showed a peak in April 2020 among LTC residents but not in the community. This probably reflects the intrinsic vulnerability of LTC facilities that concentrated COVID-19 fatality cases in 2020 [29]. It is not possible to investigate the impact of stricter restrictions in Finland in this study, but the different national strategies reflect the lack of harmonized European guidelines and policies for LTC during the first months of the pandemic [30].

The COVID-19 pandemic brought many of the complexities of organizing care for older people, including care coordination and care transitions, to the fore [7,31]. Sweden’s initial response to the pandemic has been criticized for a lack of coordination between national care authorities and under-resourced LTC [12,32]. Some of our results might be linked to the different restrictions in Finland and Sweden. That entry into and exit rates from LTC facilities changed more in Finland might indicate that Finland had a more active early response to the pandemic. However, shutting down short-term respite care could have increased the stress and burden of informal caregivers.

Cross-national comparison of Covid-19 related outcomes has been proven difficult in general [33]. In this study, we provide descriptive evidence from two countries with similar welfare systems both having large sectors for formal LTC. Although it is not possible to isolate specific factors contributing to the different patterns of LTC use in the respective countries, our results point towards some areas for future research. The use of short-term placement seems to be different across the countries; this might also be related to organizational aspects of long-term care, such as staffing, the level of medical care available, readiness to act in relation to new health threats, which are also likely important factors for responding to pandemics in LTC. The lack of information about organizational aspects is indicative of the more limited nature of the LTC registers compared with other national health registers. An important lesson from this pandemic is that the content and quality of LTC registers should be improved to prepare for future health crises [34]. This would also improve the possibility of assessing the effectiveness of other public health interventions, such as vaccination and remote healthcare delivery in the LTC setting, also important to inform strategies for mitigating the impact of future pandemics.

### Strengths and limitations

The main strength of this study is the use of large nationwide data with complete information on LTC use and hospital admissions from two countries. The data are recorded in a similar structure in both countries, and limited harmonization has been conducted for this descriptive study. Our study highlights the strength of using nationwide register data with a common protocol to conduct a comparative study between two countries. Several limitations should be highlighted. First, the lack of previous comparisons of LTC use using register data between the countries limits the possibility of isolating changes due to the pandemic to explain the differences between the two countries. For example, compositional characteristics of LTC residents beyond age and sex, and clear definitions of the placements covered by LTC, represent a knowledge gap. By including the two years before the pandemic, we provide some novel information about this, but a full-scale comparison falls outside the scope of this study. Second, LTC data in both countries are examples of routine administrative data which is not collected for research primarily. For example, some municipalities have not consistently reported data to the Swedish Social Services register [20]. In a sensitivity analysis, we excluded persons living in these municipalities, but this did not alter the rates presented in the main analyses. Additionally, home help services are important to understand the general use of care for older people, but registration of these services is difficult to compare across the two countries. Internationally, there is large variation in LTC systems in relation to infrastructure, funding mechanisms and legal frameworks. This limits the generalizability of our results to primarily other countries without needs-assessed and publicly funded LTC systems. Third, only the first year of the COVID-19 pandemic is covered by our data, which relates to the first and part of the second wave. Most COVID-19 cases in Finland came during the latter part of the pandemic and are not covered by this study.

## Conclusion

In addition to the widely different levels of COVID-19 mortality during the first year of the pandemic, we found a larger decrease in LTC use in Finland versus Sweden, and a smaller decrease in hospital admissions among LTC residents in Finland than in Sweden. The decrease in hospitalizations in both countries, as well as the decrease in short-term care in LTC in Finland, may have led to unmet care needs. Conversely, these actions may have been beneficial for the older population at large. This study highlights the usefulness of nationwide register data on LTC use for assessing the health consequences of the COVID-19 pandemic. Integrating additional information about organizational aspects of LTC in national data is important for research and can contribute to the development of future pandemic preparedness.

## Supplemental Material

sj-docx-1-sjp-10.1177_14034948241235730 – Supplemental material for Long-term care use, hospitalizations and mortality during COVID-19 in Finland and Sweden: A nationwide register-based study in 2020 Supplemental material, sj-docx-1-sjp-10.1177_14034948241235730 for Long-term care use, hospitalizations and mortality during COVID-19 in Finland and Sweden: A nationwide register-based study in 2020 by Pierre-Olivier Blotière, Géric Maura, Jani Raitanen, Jutta Pulkki, Leena Forma, Kristina Johnell, Mari Aaltonen and Jonas W. Wastesson in Scandinavian Journal of Public Health
