# Supplementary material for: Long-term care use, hospitalizations and mortality during COVID-19 in Finland and Sweden: A nationwide register-based study in 2020
Source: Scand J Public Health. 2024 Mar 13;52(3):345–53. doi: 10.1177/14034948241235730 (PMC11067386; doi:10.1177/14034948241235730)
Supplement: sj-docx-1-sjp-10.1177_14034948241235730 – Supplemental material for Long-term care use, hospitalizations and mortality during COVID-19 in Finland and Sweden: A nationwide register-based study in 2020 [file sj-docx-1-sjp-10.1177_14034948241235730.docx]

**Supplementary material**

**Supplementary Table 1**. Number of individuals included in the Swedish study population according to the exclusion criteria used regarding the consistency in the reporting of LTC use by the Swedish municipalities

|  | 2018 | 2019 | 2020 |
| --- | --- | --- | --- |
| No exclusion | 1,443,620 | 1,484,210 | 1,523,098 |
| Exclusion criteria 1 (main analysis) | 1,371,685 | 1,410,306 | 1,447,213 |
| Exclusion criteria 2 | 1,328,833 | 1,366,351 | 1,402,197 |
| Exclusion criteria 3 | 1,250,574 | 1,285,595 | 1,318,950 |

LTC = long-term care

Exclusion criteria 1: exclusion of the municipalities of Linköping, Söderköping, Örebro, Ånge, Hofors, Sorsele, Simrishamn, Köping and Sundsvall (main analysis).

Exclusion criteria 2: exclusion criteria 1 + exclusion of all municipalities for which the monthly number of LTC residents is at least 50% lower or higher than the mean between January 2018 and December 2019 at least once.

Exclusion criteria 3: exclusion criteria 1 + exclusion of all municipalities for which the monthly number of LTC residents is at least 25% lower or higher than the mean between January 2018 and December 2019 at least once.

**Supplementary Figure 1.** Unstandardized monthly outcomes


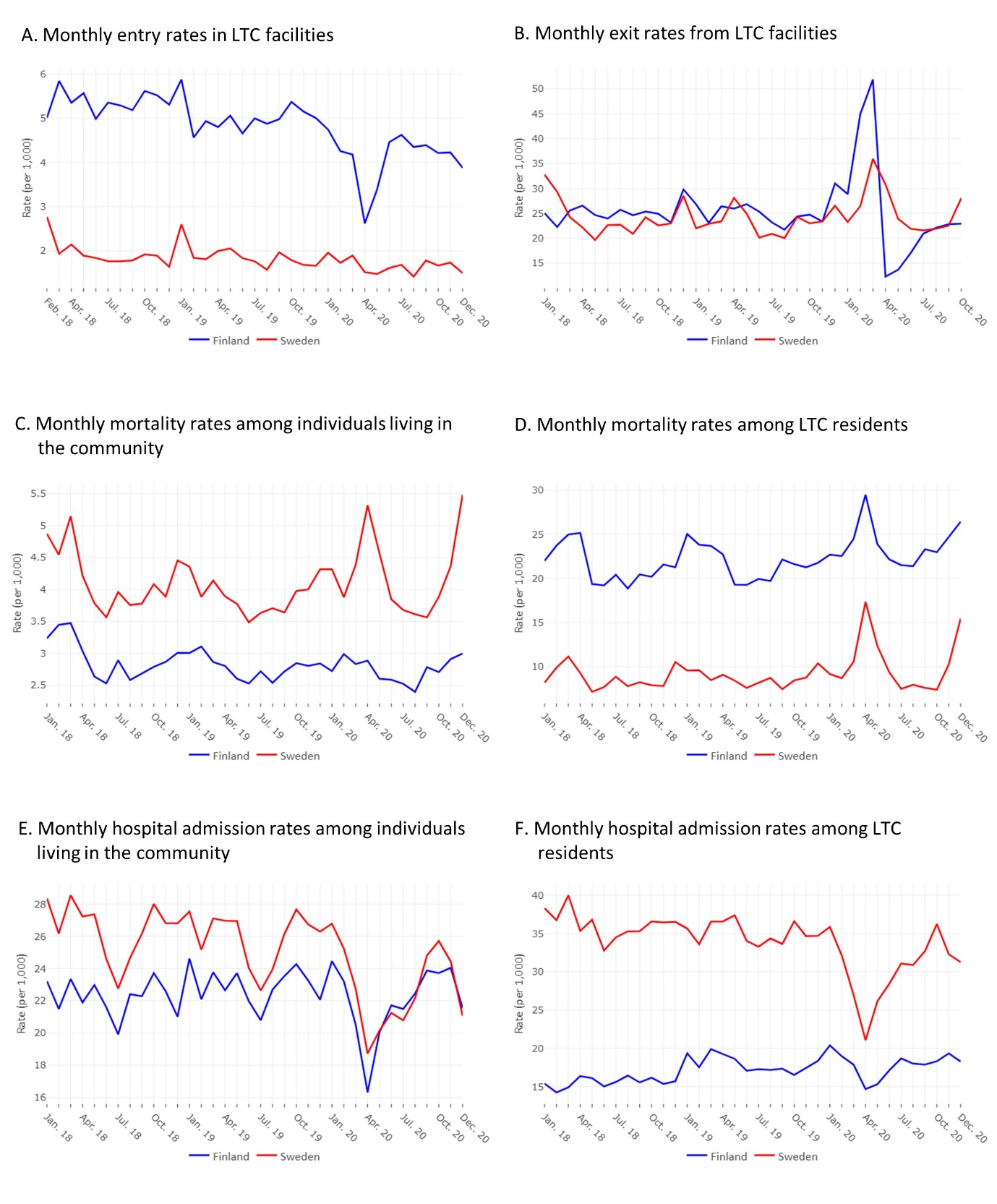
LTC = long-term care

*As no data on LTC use is available before 2018-01-01 for Finland and after 2020-12-31, outcome A was calculated from February 2018 and outcome B was calculated until October 2020.

**Supplementary Figure 2.** Sensitivity analyses regarding the definition of entry into/exit from a LTC facility and hospital admission among LTC residents.


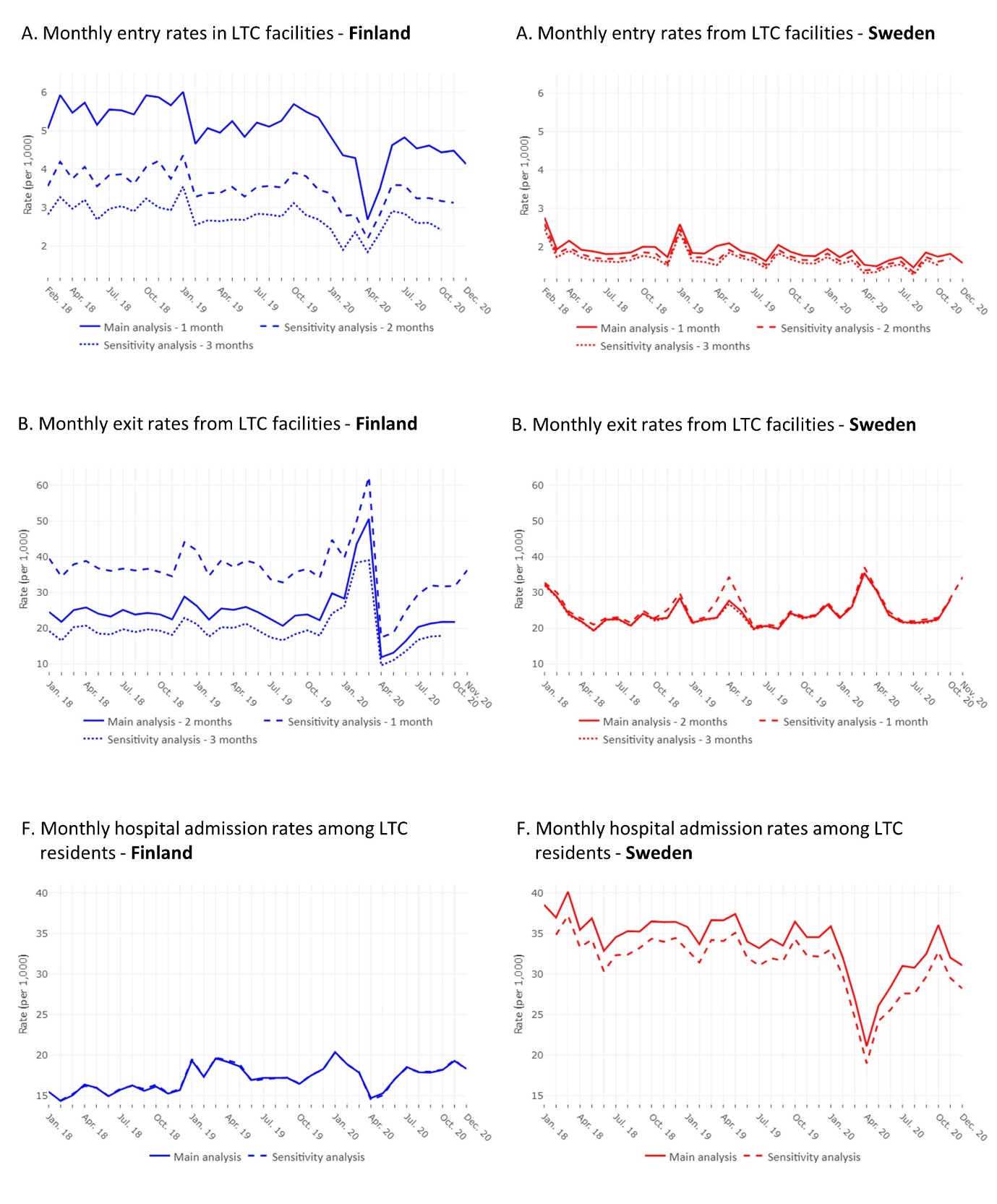


LTC = long-term care

Fig. A.

Main analysis - 1 month: entry into a LTC facility was defined as registration in a LTC facility in the current months.

Sensitivity analysis - 2 months: entry in a LTC facility was defined as registration in a LTC facility both in the current month and in the following month (as no data on LTC use is available after 2020-12-31, this sensitivity analysis was performed until November 2020).

Sensitivity analysis - 3 months: entry in a LTC facility was defined as registration in a LTC facility in the current month and in the following 2 months (as no data on LTC use is available after 2020-12-31, this sensitivity analysis was performed until October 2020).

Fig. B.

Main analysis - 2 months: exit from a LTC facility was defined as no registration in a LTC facility in the following 2 months (as no data on LTC use is available after 2020-12-31, the main analysis was performed until October 2020).

Sensitivity analysis - 1 month: exit from a LTC facility was defined as no registration in a LTC facility in the following month (as no data on LTC use is available after 2020-12-31, this sensitivity analysis was performed until November 2020).

Sensitivity analysis - 3 months: exit from a LTC facility was defined as no registration in a LTC facility in the following 3 months (as no data on LTC use is available after 2020-12-31, this sensitivity analysis was performed until September 2020).

Fig. F.

Sensitivity analysis: LTC residents were defined as individuals registered in a LTC facility in the given month and the previous month (as no data on LTC use is available before 2018-01-01 for Finland, this sensitivity analysis was performed from February 2018).

**Supplementary Figure 3.** Monthly outcomes according to stricter exclusion criteria regarding the consistency in the reporting of LTC use by the Swedish municipalities


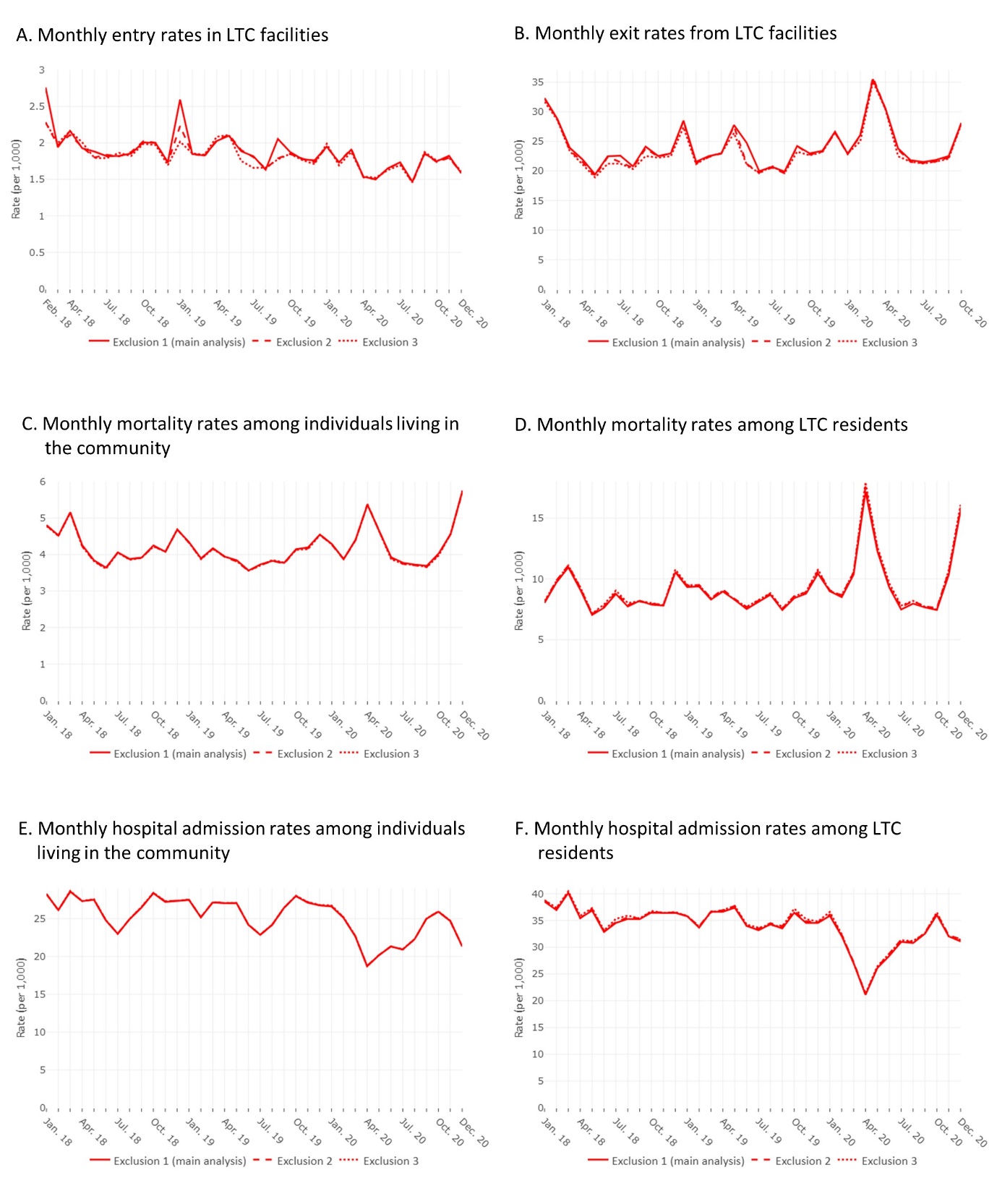


LTC = long-term care

Exclusion criteria 1: exclusion of the municipalities of Linköping, Söderköping, Örebro, Ånge, Hofors, Sorsele, Simrishamn, Köping and Sundsvall (main analysis)

Exclusion criteria 2: exclusion criteria 1 + exclusion of all municipalities for which the monthly number of LTC residents is at least 50% lower or higher than the mean between January 2018 and December 2019 at least once.

Exclusion criteria 3: exclusion criteria 1 + exclusion of all municipalities for which the monthly number of LTC residents is at least 25% lower or higher than the mean between January 2018 and December 2019 at least once.
